# Preexisting vs. de novo antibodies against SARS-CoV-2 in individuals without or with virus infection: impact on antibody therapy, vaccine research and serological testing

**DOI:** 10.1186/s41231-021-00093-2

**Published:** 2021-07-01

**Authors:** Kar Muthumani, Ziyang Xu, Moonsup Jeong, Joel N. Maslow, Vaniambadi S. Kalyanaraman, Alagarsamy Srinivasan

**Affiliations:** 1 GeneOne Life Science, Inc, Seoul, 07335 South Korea; 2 Perelman School of Medicine at the University of Pennsylvania, Philadelphia, PA 19104 USA; 3 Department of Medicine, Morristown Medical Center, Morristown, NJ 07960 USA; 4 IDC Diagnostics, Rockville, MD 20852 USA; 5 NanoBio Diagnostics, West Chester, PA 19382 USA

## Abstract

The causative agent of the ongoing pandemic in the world is SARS-CoV-2. The research on SARS-CoV-2 has progressed with lightning speed on various fronts, including clinical research and treatment, virology, epidemiology, drug development, and vaccine research. Recent studies reported that sera from healthy individuals, who were confirmed negative for SARS-CoV-2 by RT-PCR method, tested positive for antibodies against spike and nucleocapsid proteins of SARS-CoV-2. Further, such antibodies also exhibited neutralizing activity against the virus. These observations have prompted us to prepare a commentary on this topic. While the preexisting antibodies are likely to protect against SARS-CoV-2 infection, they may also complicate serological testing results. Another unknown is the influence of preexisting antibodies on immune responses in individuals receiving vaccines against  SARS-CoV-2. The commentary identifies the potential limitations with the serological tests based on spike and nucleocapsid proteins as these tests may overestimate the seroprevalence due to cross-reactive antibodies. The inclusion of tests specific to SARS-CoV-2 (such as RBD of spike protein) could overcome these limitations.

## Introduction

Zoonotic transmission has been documented with many virus families, including retroviruses and coronaviruses [1–3]. Regarding the latter group, multiple outbreaks representing three different coronaviruses have been noted in the past eighteen years [4]. Severe acute respiratory syndrome coronavirus (SARS-CoV) was involved in an outbreak in 2002 in Guangdong Province, China [5]. This virus ultimately caused around 8000 infections in 26 countries, with a total of 774 deaths. The case-fatality rate was close to 10% (WHO-Summary of probable SARS cases with onset of illness from November 2002 to July 2003. 2004). Ten years later, Middle east respiratory syndrome coronavirus (MERS-CoV) was shown to be the cause of an outbreak in Saudi Arabia in 2012. MERS-CoV infected a total of 2494 individuals in 27 countries and resulted in 858 deaths. The case-fatality rate was around 35% (WHO-Middle East Respiratory Syndrome Coronavirus (MERS-CoV). The latest coronavirus associated with an outbreak is designated as Severe acute respiratory syndrome coronavirus-2 (SARS-CoV-2). This virus was first identified in Wuhan, Hubei Province, China, in December of 2019 [5–7]. SARS-CoV-2 has now spread to more than 230 countries in the Globe and was declared as a pandemic in March of 2020 by WHO. As of May 26, 2021, there have been 167,423,479 confirmed cases and 3,480,480 deaths globally. In the US alone, the number of infections and deaths as of the same date was 33,942,991 and 605,236, respectively (https://coronavirus.jhu.edu/). This virus is highly transmissible due to specific genetic features that promote higher affinity binding to human receptors, as compared to SARS-CoV and MERS-CoV. Moreover, subsequent mutations in the virus that have arisen during the pandemic, including a specific mutation in the S protein residue 614 from aspartic acid (D) to glycine (G), have increased viral transmission potential [8–12]. In addition to these three pathogenic coronaviruses, four less virulent viruses in the coronavirus family are associated with self-limited upper respiratory tract infections in humans. Of these, HCoV-OC43 and HCoV-229E were identified in the mid-1960s [13, 14]. HCoV-NL63 and HCoV-HKU1 have been isolated in 2004 and 2005, respectively [15, 16].

Currently, a combination of five viruses (OC43, 229E, NL63, HKU1 and SARS-CoV-2) are circulating in the population worldwide. In addition, two viruses (SARS-CoV and MERS-CoV) have been associated with limited outbreaks that were geographically contained. This scenario poses key questions pertinent to understanding the pathogenesis and therapeutic options for SARS-CoV-2. This is based on the notion that prior infections with closely related viruses in people could have a tremendous influence on the infection and disease course of other viruses. There are multiple questions related to the impacts of infections by closely related viruses: i) whether prior infection with a related non-pathogenic coronavirus may provide some level of protection towards subsequent infection by another member of the same virus family due to the preexisting immunity elicited by homologous genes; ii) conversely, whether prior infection with a non-pathogenic coronavirus has the potential to induce immune activation worsening the clinical course of disease or whether, the *in vitro* phenomenon of antibody-dependent enhancement of virus infection, shown for other viruses [17–25] could occur *in vivo* to accelerate viral replication; iii) or whether co-infection with different coronaviruses could increase pathogenicity resulting from either additive or synergistic effects; additionally, from a diagnostic perspective, iv) whether antibody responses against a prior non-pathogenic coronavirus could result in false-positive serological tests meant to screen for targeting SARS-CoV-2. In this regard, Sagar *et al*. (2021) reported that individuals infected with related common cold coronaviruses exhibited less severe COVID-19 disease, suggesting preexisting immune responses may mitigate the severity of the disease [26].

The work carried out by investigators on different viruses over the years have provided answers to the questions mentioned above [26–29]. In the context of the current pandemic, we are facing a similar situation with coronaviruses as there are multiple viruses known to infect humans. The purpose of the commentary is to connect the ongoing serological studies on COVID-19 patients to the following areas: i) influence of preexisting immunity on the extent and breadth of immune responses in individuals with SARS-CoV-2 infection in comparison to individuals who received vaccines; ii) cross-reactive antibody responses due to shared epitopes among related viruses; iii) cross-reactive humoral responses due to molecular mimicry; iv) issues with prevalence studies due to cross-reactive antibodies.

## Pre-existing antibodies in the individuals against SARS-CoV-2 in the absence of virus infection

In the area of serological testing, sera collected from pre-pandemic days have generally been used as controls. However, some sera in this group showed high reactivity towards spike and nucleocapsid proteins of SARS-CoV-2. It was suggested that the cross reactivity observed may likely be due to the individuals’ exposure to the related coronaviruses. This has been the subject of several studies [30–35]. Ng *et al*. recently reported that healthy individuals, who were confirmed as negative for SARS-CoV-2 infection by RT-PCR, have been shown to harbor cross-reactive antibodies against the spike protein of SARS-CoV-2 by a flow cytometry-based assay [32]. The antibodies found in healthy individuals were primarily of IgG isotype and are more prevalent among children than adults. The antibodies noted in healthy individuals were primarily reactive against the spike protein S2 subunit that mediates viral fusion between the viral and host cell membranes and has greater level of sequence conservation than the S1 subunit involved in cellular receptor binding. The cross-reactive antibodies in healthy individuals were found to exhibit neutralizing activity against live SARS-CoV-2 virus and pseudotype virus assays, thus suggesting possible protective or disease ameliorating potential [36–38]. The antibodies present in the sera of SARS-CoV-2 infected patients showed IgG, IgM, and IgA antibodies against spike protein. Further, antibodies showed cross-reactivities towards other members such as SARS-CoV and OC43 but not with NL63 and 229E [39]. Majdoubi *et al*. (2021) reported that about 90% of uninfected adults showed cross-reactive antibodies against spike protein, receptor-binding domain (RBD), N-terminal domain (NTD), or nucleocapsid protein [40]. Peptide array covering the proteins encoded by SARS-CoV-2 genome showed reactivity specific to spike and to conserved nonstructural viral proteins. The preexisting immunity in the form of antibodies (IgA, IgM, and IgG) in milk in individuals negative for virus infection is shown to be related to the infections with OC43 and 229E. While IgG was specific to S2, both IgA and IgM were reactive to S1 and S2 of spike protein [41]. The profiling of sera from 43 individuals for immune responses against different coronaviruses indicated that preexisting immunity was directed to NL63 and HKU1. This status had no influence on the antibody response against SARS-CoV-2 [42]. Similarly, the findings of Simula *et al*. (2020) showed that antibodies against NL63 have the potential to cross-react against SARS-CoV-2 spike protein [43]. The preexisting humoral responses against SARS-CoV-2 were also reported to be against N and spike proteins [44, 45]. The presence of preexisting antibodies in healthy individuals is further evidence that intravenous immunoglobulin (IVIg) derived from healthy donors showed positivity to SARS-CoV-2 antigenic epitopes likely resulting from epitopes of common cold coronaviruses [46].

Shrock *et al*. (2020) also recently reported cross-reactive antibodies against the ORF1 protein in the sera from pre-COVID-19 healthy controls using virscan approach involving peptides representing genomes of several members of the coronavirus family including SARS-CoV-2 [47]. In this study, antibodies against spike protein were not detected, possibly be due to the assay format. Again, whether such antibodies mitigate disease due to SARS-CoV-2 is unknown. It may require population-based studies that investigate stored sera (such as from blood banks) to determine the relative risk for infection and severe disease as a function of antibody titers and target antigens.

Importantly, it would be of interest as to whether protective epitopes or antigenic regions could be defined through: i) elucidating the nature of epitopes targeted by pre-existing antibodies in the healthy individuals without SARS-CoV-2 infection; ii) comparing amino acid sequences of the potential epitopes among human coronaviruses for homology, and with respect to viral variants appearing during the pandemic iii) determining the difference in the repertoire of humoral responses induced by modified recombinant spike proteins in comparison to the unmodified spike proteins. However, determination of the relevance of preexisting sero-reactivity necessitates determining whether reactive serological tests represent actual exposure to SARS-CoV-2 infection or arise from molecular mimicry to other Coronaviruses.

## Cross-reactivities resulting from shared epitopes?

The complete genome sequence of SARS-CoV-2 became available in the middle of January 2020. The genome wide pairwise comparison shows 79.6% and 50% homology to SARS-CoV and MERS-CoV, respectively [48]. The homology at the spike protein level between SARS-CoV-2 and SARS-CoV shows 75%, 64%, 90%, 51%, and 50% for full length protein, S1 RBD, S2 fusion domain, S1 NTD, and S1 RBM, respectively [12, 49]. Based on the information available regarding experimentally verified epitopes in SARS-CoV, Ahmed *et al*. analyzed SARS-CoV-2 sequence for predicting potential epitopes [48] revealing 49 epitopes which are identical between SARS-CoV-2 and SARS-CoV. Of the 49 epitopes, 23 are located in the S protein, and 20 of the 23 epitopes fall in S2 subunit region. The second-largest number of cross-reactive epitopes mapped to the N protein (22 epitopes), while the last 4 were within the M protein. Grifoni *et al*. analyzed and predicted the dominant epitope regions in SARS-CoV-2 based on SARS-CoV and other related viruses [50]. The predicted conserved epitopes in the SARS-CoV-2 S protein regions comprised of fragments at the following amino acids positions: 287–317, 524–598, 601–640, 802–819 and 888–909; and the epitopes in N protein were located at the following positions 42–62, 153–172 and 355–401. Forcelloni *et al*. reported on the conservation of epitopes in spike and nucleoprotein of coronaviruses [51]. The C-terminal region of the spike protein and the nucleoprotein RNA binding (41–186 aa) and dimerization (258–361 aa) domains harbor B and T cell epitopes which may provide protection against other members of coronaviruses [51]. Therefore, antibodies against these conserved epitopes may result in cross-reactivity in antibody assays.

## Presence of antibodies against S protein in individuals without SARS-CoV-2 infection: result of potential molecular mimicry

The reports of autoimmune and inflammatory diseases in SARS-CoV-2 infected patients suggested that potential homologous regions in SARS-CoV-2 protein and cellular proteins may lead to such conditions. Molecular mimicry is a well-established phenomenon when proteins encoded by distinct or unrelated genes share similar structures [52]. Such structures may be due to conformational features or homology at the primary amino acid sequence level. Based on this, there is a possibility that cross reactive antibodies in healthy individuals without SARS-CoV-2 infection could be the result of molecular mimicry with other coronaviruses and cellular genes. Several groups reported that a significant homology was noted between the macrodomain of SARS-CoV-2 nsp3 and ADP-ribose glycohydrolase MACROD1, MACROD2, protein mono-ADP-ribosyltransferase PARP14 and PARP 9 [53]. Hwa *et al*. (2007), based on the analysis of homology between spike protein of SARS-CoV and human cellular proteins, provided evidence in support of molecualr mimicry  [54]. This analysis identified residues 199–254, 658–715, 893–951, and 1127–1184 exhibited homology with hydroxy acid oxidase, human golgi autoantigen, Angrgm 52, and pallidin, respectively. The peptides derived from these regions of spike (D01, aa 199–210; D07, aa 927–937; D08, aa 942–951) showed reactivity towards the sera from SARS-CoV patients. Further, it was shown that hyperimmune sera against D08 and D07 cross reacted with human A549 cells and D10 (aa 490–502) cross reacted with bradykinin [54]. These studies suggest that homology region in cellular protein may lead to the induction of autoantibodies in healthy individuals upon infections by coronaviruses. These autoantibodies are likely to be recognized by the S2 domain of spike protein of SARS-CoV-2 [55]. Vojdani *et al*. (2021) reported that 28 out of 55 tissue antigens showed reactivities towards monoclonal antibodies (spike and nucleoprotein) and polyclonal antibodies (E and M) against SARS-CoV-2 proteins [56]. It was further noted that the amino acid sequence similarities between SARS-CoV-2 and cellular proteins including Mitochondrial M2, F-actin and TPO were the source of reactivities. Recently, Qiang *et al*. (2021) showed high homology between a segment (YNYLYR) in RBM and the epitope sequence NDALYEYLRQ of several monoclonal antibodies against tetranectin. The two tetranectin monoclonal antibodies were shown to bind RBM with a KD of 17.4 and 62.8 nM, respectively [57].

## Impact on antibody therapy and vaccine research

Monoclonal antibody therapy or convalescent plasma therapy is currently used to treat individuals infected with SARS-CoV-2. The goal is to competitively interfere with the binding between the spike protein S1 domain and the ACE2 receptor [58–61]. It is also possible that antibodies to S2 subunit of the spike protein could also interfere with the infection process through blocking at the level of viral and cell membrane fusion. It was recently reported that polyclonal antibodies present in convalescent COVID-19 patients showed neutralizing activity against the virus. This has been the basis for the use of convalescent plasma for the treatment of patients with severe COVID-19 disease [62, 63]. This approach has been granted emergency use authorization in several countries. Interestingly, healthy individuals with possible exposure to common cold coronaviruses may also contain antibodies with neutralizing activity [32, 64] potentially expanding the pool of “therapeutic” serum donors.

While the potential therapeutic benefit of convalescent serum and, by definition, neutralizing monoclonal antibodies targeting S1 protein is based on interfering with viral adherence to the ACE2 receptor, the studies by Ng *et al* also suggest that S2 protein reactivity in convalescent sera should be evaluated as a predictor for clinical success. The monoclonal antibodies targeting the S2 subunit should be evaluated for neutralizing activity and therapeutic benefit [32]. Moreover, S2 is likely to require a greater degree of sequence conservation among virus variants, targeting this subunit may avoid potential viral escape through mutation in the S1 binding domain. The recent demonstration of cellular immune responses in patients who recovered from COVID-19 suggest that induction of a combination of humoral and cellular immune responses may be useful for optimal protective effect [65–67]. This is also evident in the studies with mRNA, vectored viral and inactivated viral vaccines [68, 69].

In light of preexisting immunity against SARS-CoV-2 in the general population, answers to the following questions would benefit vaccine studies: i) are there safety concerns for administering vaccines to individuals with preexisting immunity?; ii) do the individuals with preexisting immunity show elevated levels of immune responses in comparison to naïve individuals?; iii) do the individuals with preexisting immunity require only one dose of vaccine instead of the required two doses regimen in the case of approved Pfizer-BioNTech and Moderna mRNA vaccines? Assis *et al*. (2021) reported that individuals with prior exposure to the virus generated a high antibody response to spike protein in comparison to the individuals without prior exposure. It is not known whether individuals infected with related viruses generate a similar response [70].

## Impact of antibody cross-reactivity on serology tests

**Fig. 1:**
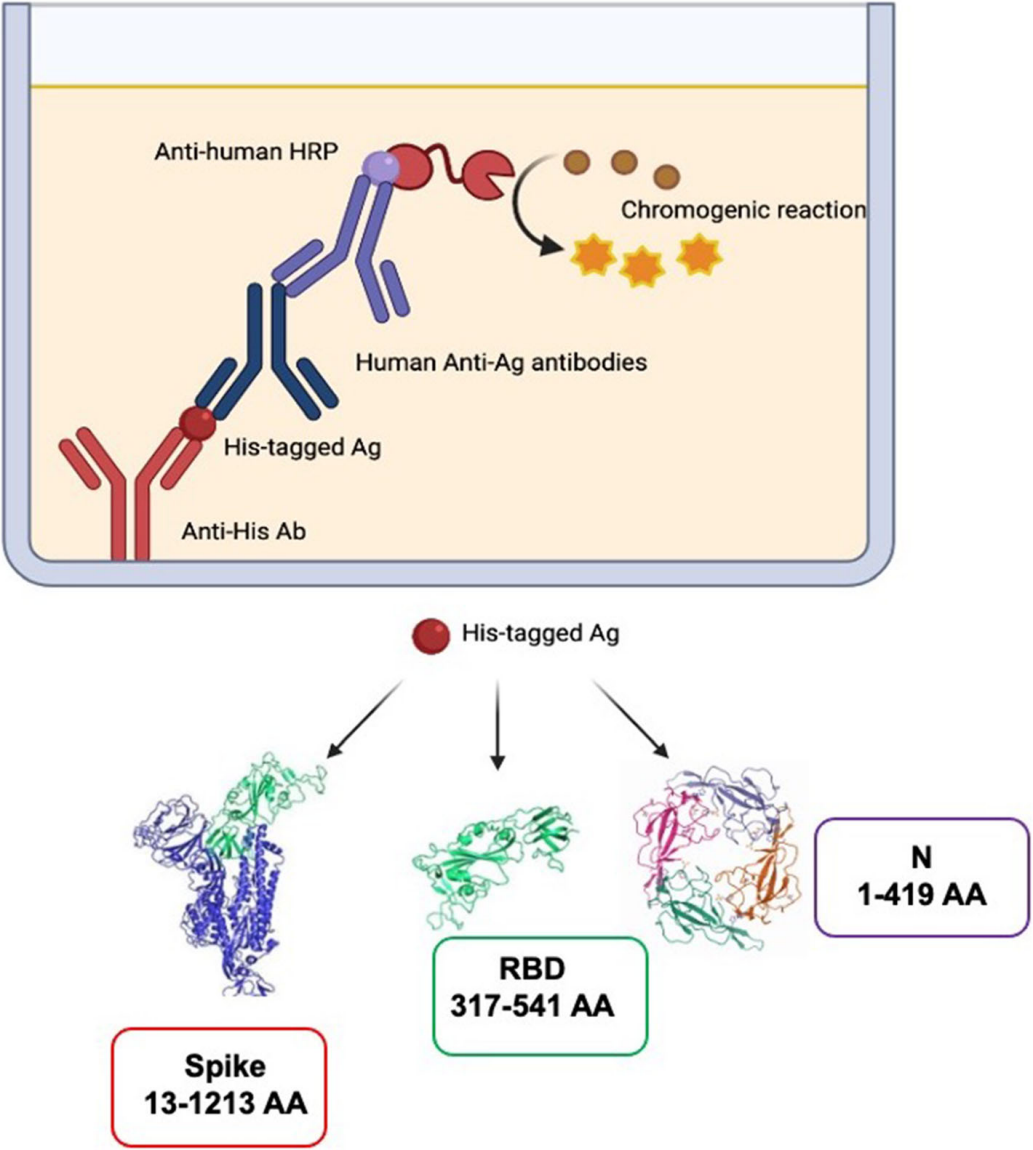
Serological assays using various His-tagged SARS-CoV-2 antignes. The boundaries of the substrates (S, RBD and N) are indicated by the amino acid numbers from N- and C-termini, respectively

The tests that examine antibodies as biomarkers have been generally carried out in the groups including virus infected individuals with mild, moderate and severe disease symptoms, convalescing patients, asymptomatically infected individuals, and general population. However, the validation of antibody tests utilized sera from healthy individuals as negative controls which were collected during pre-pandemic days but recent publications have shown the presence of cross-reactive antibodies against the ORF1 and spike proteins of other Coronaviruses [32, 47] in addition to the highly homologous core N protein. This is not surprising since the members of the coronavirus family infecting humans are closely related. Several investigators have reported on the appearance of antibodies to SARS.Cov2 proteins in the blood/ sera after the onset of symptoms in SARS-CoV-2 infected individuals [67, 71, 72]. The general trend has been that IgM and IgA antibodies appear in the first week after the onset of symptoms and IgG antibody appears in the second week, though there are also reports on its appearance in the first week [9, 73–77]. Due to cross-reactivity, tests measuring antibodies may not serve as tests for the diagnosis of COVID-19. It has been shown, however, that serological tests serve as adjunct tests for the diagnosis in situations where patients with the designated symptoms are negative for SARS-CoV-2 by RT-PCR [78–80]. Serological antibody tests are important for evaluating disease incidence and seroprevalence evaluation in the population. The prevalence rates were found to be in the range of 1-5% depending on the country and location selected for analysis [10, 67]. Specifically, viral structural proteins such as spike and N have been used as substrates for evaluation of antibodies (Fig. 1). While results could be readily generated from these assays (ELISA and immunochromatography), their interpretation may not be as straightforward if the individuals tested had previously been exposed to a related virus [67].

**Fig. 2:**
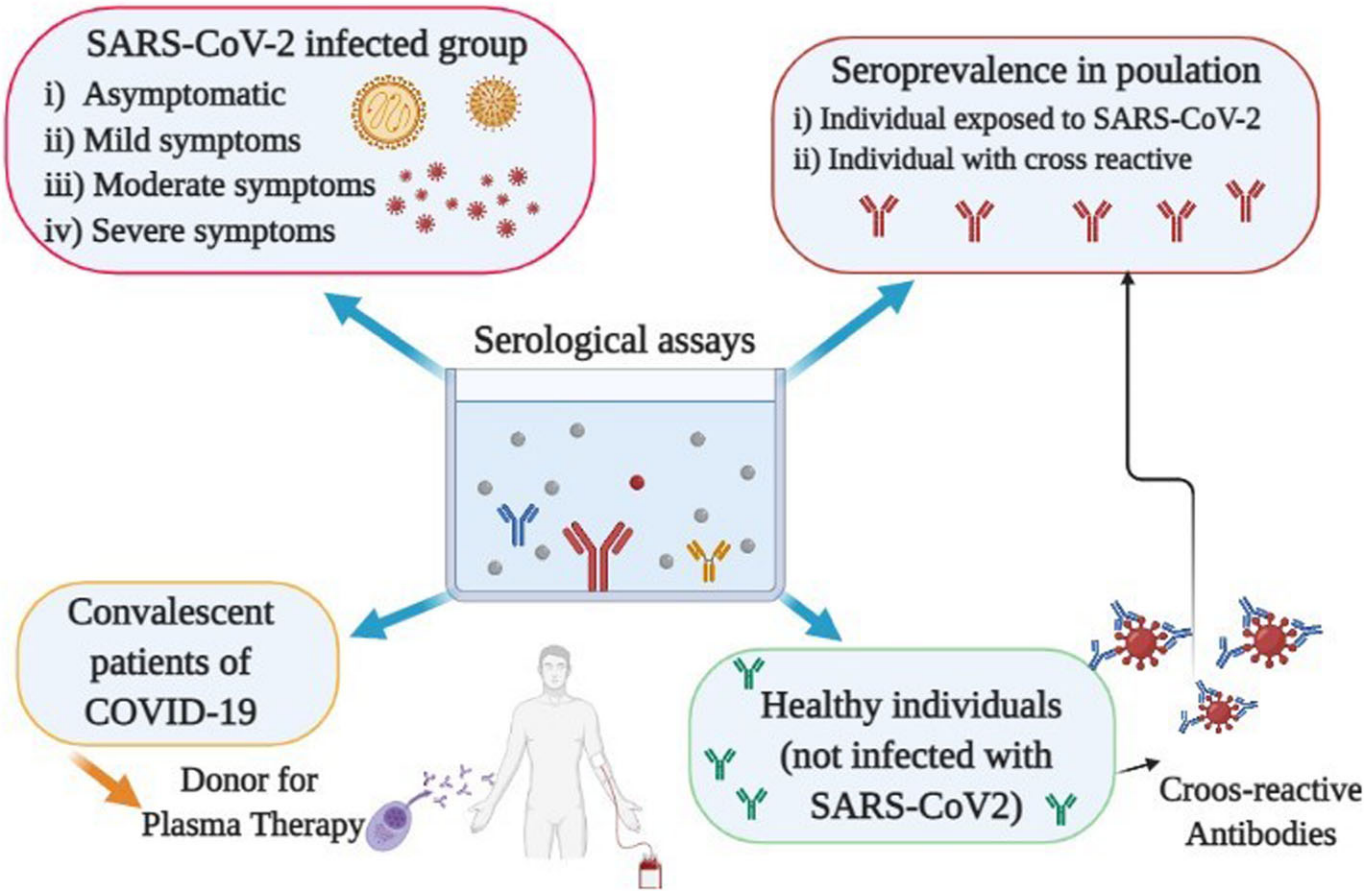
Schematic diagram of the serological tests used for evaluation of individuals with or without SARS-CoV-2 infection

For example, if an individual is exposed to common cold coronaviruses, the antibodies elicited in response to such infections are likely to cross react with SARS-CoV-2 encoded proteins due to homology between these viruses. In support of this, recent studies demonstrated preexisting antibodies against SARS-CoV-2 proteins in healthy individuals without SARS-CoV-2 infection [32]. It should be mentioned that such preexisting humoral responses may lead to overestimation of the prevalence rates in the population (Fig. 2). Though SARS-CoV and MERS-CoV infections have been confined to fewer countries and have not been actively in circulation, the situation with common cold human coronaviruses is different. Currently, there are commercial kits in the market that measure antibodies in the sera using only the N protein as the substrate. Hence, the incidence and prevalence estimation studies should be carried out with antigenic substrates that have the least homology with related coronaviruses.

## Summary

In summary, several studies have reported that individuals may have pre-existing antibodies to recently emerged SARS-CoV-2 prior to the outbreak due to infections with related common cold coronaviruses, homology in the primary amino acid sequences of proteins encoded by these viruses, and molecular mimicry in the important antigenic targets. On the one hand, such antibodies may prove to be extremely beneficial by providing pre-existing protective immunity to the hosts when they bind to the conserved neutralizing epitopes on the S2 domain of the spike protein. On the other hand, they can also represent a challenge through potential antibody-mediated enhancement, which can exacerbate the severity of the infection by the emerging coronavirus, but this has not yet been well-described in humans in the case of SARS-CoV-2. Additionally, these pre-existing antibodies may serve as a confounding factor and render it hard to study true prevalence of infection in a population through the use of routine serological assays. Future studies to further characterize the epitope-specificity and neutralization potential of these cross-reactive antibodies are clearly of great importance to mitigate the current COVID-19 pandemic and to prevent possible future coronavirus outbreaks.

## Data Availability

The commentary is based on the published literature on SARS-CoV-2. The authors confirm that the data supporting the findings of this study are available within the article.
